# Personality, Coping Strategies, and Mental Health in High-Performance Athletes During Confinement Derived From the COVID-19 Pandemic

**DOI:** 10.3389/fpubh.2020.561198

**Published:** 2021-01-08

**Authors:** Federico Leguizamo, Aurelio Olmedilla, Antonio Núñez, F. Javier Ponseti Verdaguer, Verónica Gómez-Espejo, Roberto Ruiz-Barquín, Alexandre Garcia-Mas

**Affiliations:** ^1^GICAFE (Research Group of Sports Sciences), University of the Balearic Islands, Mallorca, Spain; ^2^Department of Personality, Evaluation and Psychological Treatment, University of Murcia, Murcia, Spain; ^3^Department of Evolutive and Educational Psychology, Autonomous University of Madrid, Madrid, Spain

**Keywords:** sports psychology, personality, high-performance athletes, coping, stress, COVID-19, confinement

## Abstract

The COVID-19 outbreak has affected the sports field unprecedentedly. The emergency alert has deprived athletes of training in a suitable environment, as they are faced with cancellations of relevant events in their sports careers. This situation can cause stress levels and other emotional disorders similar to those experienced by athletes during periods of injury. Since the relationship between psychological factors and sports injuries is well-studied, the Global Psychological Model of Sports Injury (MGPLD) is applied to this historical situation for athletes. The purpose of this study was to analyze the relationships between perfectionism and trait anxiety with indicators of mental health (mood, depression, state anxiety, and stress) in high-performance athletes during confinement due to the COVID-19 pandemic, as well as to explore the coping strategies that athletes have applied and whether they are perceived as useful for managing negative emotional states. A cross-sectional study was conducted through online questionnaires during April 2020, adapting the Psychological Assessment Protocol of the High-Performance Sports Center of Murcia (Spain), to assess the psychological effects of confinement in a cross-cultural sample of 310 athletes (141 women and 169 men) from different countries in Europe, Asia, and America, and from diverse sports disciplines. The protocol comprised six instruments that test perfectionism, trait anxiety, mood states, stress, depression, coping strategies, and sleep. It was answered online via Google Forms. The results show that maladaptive perfectionism was related to all the indicators of athletes' mental health. However, athletes' levels of anxiety, stress, and depressive symptoms are relatively low, and the use of coping strategies such as cognitive restructuring and emotional calm was associated with lower levels of negative emotional states. Besides, the Iceberg Profile, a suitable fit for the mental health model, is observed in the mood of athletes, both in men and in women, although women showed higher levels of anxiety, stress, and depression than men. A strong relationship was observed between maladaptive perfectionism and martial arts sports discipline, superior to other sports. In short, it can be concluded that high-performance athletes in the studied sample showed negative emotional state values below the expected average. Finally, the proposals for practical applications of the results collected are discussed.

## Introduction

The emergence and expansion of the COVID-19 disease have caused a worldwide pandemic, affecting the political, economic, and social stage. To stop the spread of the disease, measures of confinement taken by most governments have interrupted the daily lives of the people, impeding athletes of training in a suitable environment, which can lead to negative consequences at emotional, cognitive, and behavioral levels. For most athletes, this sudden interruption in their training schedule will lead them to set new goals during the season when it resumes. For athletes at the end of their competitive stage, it can mean putting an early and abrupt closure to their careers, which can increase unpleasant emotions during confinement.

A recent review developed to study the existing evidence on the psychological impact of quarantine on people concluded, after analyzing 24 studies, that this situation can have adverse psychological effects such as post-traumatic stress symptoms, confusion, and anger (1). Other studies also show that confinement can affect well-being by inducing, promoting, or increasing substance abuse (2).

This situation of confinement is similar to the state of injury that most athletes suffer at some point throughout their sports careers. According to the literature, a series of psychological and emotional reactions may appear during the sports injury, as a response to both the injury itself (3–6) and the rehabilitation process (7). Moreover, the return to competition after a period of rest due to sports injury and the fear of relapse (8) are also the aims of this study.

The Global Psychological Model of Sports Injury [MGPLD; (9)] proposes that variables such as motivation, competitive anxiety, psychosocial stress, and coping mechanisms influence the different moments of the injury (before, during, and after). From this perspective, we consider that the confinement situation caused by the COVID-19 pandemic can have significant psychological effects on athletes, similar to those seen in people who carry out daily tasks in confinement also evidenced in studies about sports withdrawal (partial or total) because of sports injury.

Since this particular and sudden governmental response to COVID-19 was unexpected for the majority of the population, confinement measures might have entailed situations of unwanted isolation. For this reason, we explored the literature on the psychological consequences of confinement. From all the research conducted on human confinement, we highlight studies carried out on inmates in prisons and those related to performance tasks under a particular situation of confinement, as it is in study cases of astronauts and the crew of underwater vessels. A recent study in consequences of isolation shows how people who experience this isolation in prison present clinical or subclinical symptoms of mood disorders (depression and anxiety) and other added psychological symptoms such as loss of identity and stimulatory hypersensitivity (10, 11).

In other studies, such as Haney's research on prisoners in confinement (12), two different kinds of symptoms are illustrated. On the one hand, there are symptoms related to affect such as anxiety, migraines, tiredness and apathy, sleep problems, nightmares, palpitations, loss of appetite, and dizziness. On the other hand, there is a series of symptoms that Haney defines as the psychopathological effects of solitary confinement, such as irrational anger, hypersensitivity to stimuli, confused thinking process, perceptual distortions, hallucinations, and suicidal thoughts.

Because of the situation of confinement that convicts live in prisons, some studies have developed a series of measures to prevent the impact of pandemics in prisons. In the USA, the Centers for Disease Control and Prevention has developed a checklist to face epidemics (13), and similarly, the WHO has developed a recent and specific response guide to COVID-19 (14).

In the case of astronauts and crew of underwater vessels, besides social isolation (to a greater or lesser extent), there is also an adaptation to an extreme environment, which differs greatly from the usual habitat where people face their day-to-day tasks. These exceptional conditions are characterized by a reduced space, added to the absence of gravity, and the social isolation that produces changes in perceptive (15), cognitive, and sensorimotor levels (16). Also, the absence of light can cause changes in work and rest schedules, lack of sleep or difficulty falling asleep, changes in circadian rhythms, fatigue, etc. (17). Although there might be negative psychological consequences of confinement, there might also be protective factors, such as the resilience of individuals, that can minimize its effect and improve people's well-being (18).

Another set of consequences that have been observed is that low mood caused by isolation in crew members of submarines and space stations induces a series of dysfunctions in the immune system, such as hormonal alterations that increase the predisposition to get sick as a result (19, 20). Confinement, the same as in sports injury, can have adverse effects on certain psychological variables. These effects can take place during confinement itself, or, as in sports injuries, they can lead to psychological and social consequences once the period of confinement ends and the population is able to resume daily activities (21).

Psychological factors underlying the different stages of sports injury are growing in importance because it influences different processes related to a sports injury, and the potential for improvement in relapse intervention and prevention (22). Although the confinement situation derived from COVID-19 is a historically novel phenomenon, problems derived bear some similarities to those suffered by athletes during the different stages of sports injury. Some of these implications are the interruption or limitation of sports activity, loss of autonomy, changes in the sports environment, loss of opportunities to improve sports records individually and collectively, interruption or limitation of non-related sports activities, and changes in personal and family life, including early retirement due to changes in competition schedules. Besides, larger problems such as substance abuse, social isolation, episodes of depression or anxiety, suicidal tendencies, self-esteem issues, and poorly perceived quality of sleep might be present. The latter factor is closely related to the athlete's quality of life, since poor perception of the quality of sleep can negatively influence athletes' well-being (23). In line with the conclusions of other authors regarding injuries, long periods of confinement could also stand an opportunity for personal growth and improvement of the psychological aspects of sports practice (24–27).

The current situation of confinement worldwide has led many athletes to adapt their sports training without the tools or suitable spaces to develop their training routines properly. This fact has led us to research how this unusual situation affects athletes and how they are experiencing it since all local, national, and international competitions have been canceled or postponed. It is in our particular interest to study the psychological effects, both negative and positive, that this situation can have on them.

Following the parallelism between injury and confinement, physiological and attentional changes as a consequence of stress caused by isolation can significantly influence athletes. If there is indeed a strong psychological relation with confinement and injury-perceived stress, those athletes with a higher level of stress and low self-control who do not put in place adequate coping resources would suffer a greater psychological impact once the competition resumes than those athletes with higher self-control and better competition stress management (28, 29).

The COVID-19 pandemic is unprecedented in terms of measures taken by countries and governments around the world; therefore, there are not many prior data to predict how this confinement will affect people in general and athletes in particular. For that reason, in the absence of a theoretical model of reference on which to base our hypotheses on the consequences that confinement by COVID-19 may have on athletes, we have advocated studying the parallelism of sports injuries and their psychological effects on the athletes.

Taking the Global Psychological Model of Sports Injury (9) as a starting point, we have selected those psychological variables that have been extensively studied in the field of sports injury and belong to the conceptual axis of sports injury. This axis forms a “galaxy” of factors, using the authors' metaphor, which influences the subjective experience of sports injury in particular ways.

Considering the aforementioned studies on this matter, the psychological variables selected for our study are the following: perfectionism (understood as concern over mistakes, personal standards, parental expectations, and organization), trait anxiety, state anxiety, stress and depression, mood state, coping strategies (understood as emotional calming, active planning/cognitive restructuring, mental withdrawal, seeking social support, and behavioral risk), and quality of sleep.

This study aims to analyze the relationships between perfectionism and trait anxiety with mental health indicators (mood, depression, state anxiety, and stress) in high-performance athletes during confinement due to the COVID-19 pandemic. In addition, the coping strategies that athletes have applied are explored and whether they are perceived as useful for managing negative emotional states.

## Materials and Methods

### Participants

An incidental sampling was carried out to get the data sample. Athletes taking part in the study are formally enrolled in their respective sports federations. The inclusion criterion to be part of the sampled population was to be a high-level athlete or a high-performance athlete in their respective countries. The sample consists of 310 athletes (141 women) from 18 to 49 years of age (M = 22.26 years; SD = 4.98). The final sample answered all questions included in the protocol, except for 15 athletes who did not specify which sport discipline they belong to. For this matter, the analysis of psychological variables and sports modalities was set to 295. The most frequent sport practiced for this sample was football (14.9%), followed by athletics (12.2%), martial arts (11.9%), basketball (9.8%), and rugby (8.1%).

All subjects completed the *Psychological Assessment Protocol* online, voluntarily, and in accordance with the Helsinki Agreement protocol. Furthermore, this study has been approved ethically by the University of Trás-os-Montes e Alto Douro (UTAD, Portugal) Ethical Committee, code 23/DOC20/CE/UTAD (27/06/2018), and follows the Helsinki Protocol guidelines, including the informed consent from all participants.

### Instruments and Materials

An *ad hoc* protocol was created, based on the injury protocol (30, 31) used in the Murcia High-Performance Center. This protocol assesses sociodemographic, personal, and sports data (name, age, sex, place of residence, household members, sport discipline, studies, course year, and online classes availability), and it consists of the following questionnaires.

The *Multidimensional Perfectionism Scale* [FMPS, (32)], Spanish version, adapted from the original FMPS (33), provides four subscales (instead of six, as previously suggested) for a multidimensional assessment of perfectionism: Concern over Mistakes (CM), Personal Standards (PE), Parental Expectations (PE), and Organization (O). The scale showed satisfactory reliability (Cronbach's α's = 0.87). The 35-item questionnaire is answered on a Likert-type scale from 1 (strongly disagree) to 5 (strongly agree); higher points show higher perfectionism while lower points indicate otherwise.

The *State-Trait Anxiety Inventory* [STAI-T; (34)], Spanish version, adapted from the original STAI-T (35), was used for the evaluation of competition anxiety. This scale has 20 items for assessing trait anxiety and 20 items for state anxiety. All items are rated on a four-point scale from 0 (hardly ever) to 3 (almost always). Higher scores indicate greater anxiety. The scale showed Cronbach's α's = 0.93.

The short version of *Depression, Anxiety, and Stress Scales* [DASS-21; (36)] Spanish version adapted (37) was used to measure common symptoms of depression, anxiety, and stress. This scale provides three subscales: Depression (DASS21-D), Anxiety (DAS21-A), and Stress (DASS21-S), each of them containing seven items. Each item comprises four response options scored from 0 (Does not apply to me at all) to 3 (Totally applies to me, or most of the time). The scale showed Cronbach's α's = 0.81.

The *Profile of Mood States* (38) was used in its Spanish adapted and validated version by Fuentes et al. (39). The short scale contains 29 items answered on a five-point scale from 0 (nothing) to 4 (very much). In this version, athletes reported on their mood state concerning each of the items on the instrument. The scale details five mood states: tension (α = 0.83), anger (α = 0.85), vigor (α = 0.83), fatigue (α = 0.82), and depression (α = 0.78).

The *Approach to Coping in Sport Questionnaire* [ACSQ-1; (40, 41)] was used in its Spanish version (42). This scale contains 28 items answered in Likert-type form from 1 (never) to 5 (always). The questionnaire aims to find out how often athletes use certain coping strategies in competitive situations. The five dimensions of coping assessed are emotional calming (7 items; e.g., “I tried to block negative thoughts”), active planning/cognitive restructuring (6 items; e.g., “I tried to find something positive in what happened”), mental withdrawal (6 items; e.g., “I thought there was nothing left to do, and I accepted it”), seeking social support (4 items; e.g., “I talked to someone to figure out what I could specifically do to solve the problem”), and behavioral risk (5 items; e.g., “I was constantly changing strategies”). Internal consistency coefficient was acceptable (α > 0.7).

A two-item questionnaire, created *ad hoc*, aims to assess the number of sleep hours and its quality, based on the *Sports Sleep Questionnaire* (CSD in Spanish) developed by Garcia-Mas et al. (23). The item of perceived quality of sleep is answered using a Likert-type scale from 1 (very bad) to 5 (very good). The second item, related to hours of sleep, was a numerical answer to indicate the number of hours that the subject usually sleeps.

### Procedure

The online tool Google Forms was used via online messaging platforms and email, allowing a fast and efficient distribution of the *Psychological Assessment Protocol* to a wide spectrum of individuals who met the inclusion criteria. This selection approach benefitted the heterogeneity of the sample population. A cross-cultural sample was obtained in collaboration with several sport psychology working groups from China, Mexico, Portugal, Russia, and Spain. Initially, a total sample of 414 subjects was obtained, of which 103 were underage. These subjects and outlier values (a 68-year-old man) were excluded from the analysis, resulting in a final sample of 310 subjects. The athletes in our sample answered the psychological protocol throughout the month of April 2020. Several countries had been in alarm stage for approximately 2 weeks at that time, with the consequent suspension of sports training, competitions, and other sporting events. In addition, there were still no expectations regarding the end of the confinement measures or the resumption of trainings and sports competitions. The data obtained through Google Forms were exported to a Microsoft Excel spreadsheet for their adequate categorization. A descriptive and correlational analysis of the questionnaire responses was conducted using a software package for statistical analysis in social sciences, the statistical program SPSS v 20.

### Data Analysis

The Kolgomorov–Smirnov normality test was applied in order to determine if parametric or non-parametric analysis was appropriate in this study. The results indicated that almost all variables present an abnormal distribution for this sample (*p* < 0.05), with the exception of STAI-T, POMSv, FMPSD, and ACSQ-U-CE (*p* > 0.05).

We applied a descriptive analysis, comparisons between groups and correlational analyses to the aforementioned sample. Given the data obtained from the results of the Kolgomorov–Smirnov test, the analysis of differences in means have been applied to several independent samples using the Kruskal–Wallis test, median difference analyses for two independent samples using the Mann–Whitney *U*-test, and correlational analysis using Spearman's Rho.

## Results

Table 1 shows the correlations between the ACSQ (Use/Efficacy) measurements and the STAI-T, DASS-21, POMS, FMPS, and Sleep Quality subscales. No significant correlation is shown between the different ACSQ factors (Use/Efficacy) and the hours of sleep, or between the ACSQ factors with the FMPSd factor except in ACSQUmw (*rho*=0.174; *p* < 0.01). The highest correlations are found in ACSQEcr and ACSQEem (*rho* = 0.396; *p* < 0.01), in a positive and negative direction.

**Table 1:** Spearman correlation coefficient between the ACSQ, STAI-Trait, DASS-21, POMS, FMPS, and Sport Sleep questionnaires.

	**SQ**	**SH**	**STAI**	**DASSd**	**DASSa**	**DASSs**	**POMSt**	**POMSd**	**POMSa**	**POMSv**	**POMSf**	**FMPSA**	**FMPSD**
ACSQUem	0.145^*^	0.011	−0.260^***^	−0.214^***^	−0.107	−0.138^*^	−0.086	−0.201^***^	−0.100	0.356^***^	−0.223^***^	0.332^***^	−0.060
ACSQUcr	0.124^*^	0.022	−0.288^***^	−0.365^***^	−0.159^**^	−0.219^***^	−0.138^*^	−0.271^***^	−0.131^*^	0.346^***^	−0.275^***^	0.218^***^	−0.143^*^
ACSQUmw	−0.207^***^	−0.024	−0.286^***^	0.291^***^	0.199^***^	0.264^***^	0.185^***^	0.253^***^	0.240^***^	−0.143^*^	0.186^***^	−0.083	0.187^***^
ACSQUbr	−0.046	−0.042	0.005	−0.011	−0.032	−0.036	0.049	0.074	0.060	0.228^***^	−0.025	0.207^***^	0.130^*^
ACSQUss	−0.009	−0.101	−0.023	−0.108	−0.039	0.011	0.088	0.004	0.041	0.076	0.044	0.144^*^	−0.048
ACSQEem	0.152^**^	0.072	−0.343^***^	−0.348^***^	−0.230^***^	−0.244^***^	−0.225^***^	−0.324^***^	−0.193^***^	0.372^***^	−0.299^***^	0.224^***^	−0.209^***^
ACSQEcr	0.199^***^	0.119^*^	−0.323^***^	−0.413^***^	−0.201^***^	−0.243^***^	−0.191^***^	−0.326^***^	−0.186^***^	0.386^***^	−0.277^***^	0.238^***^	−0.201^***^
ACSQEmw	−0.152^**^	−0.017	−0.197^***^	0.168^**^	0.194^***^	0.188^***^	0.177^**^	0.195^***^	0.232^***^	−0.059	0.109	−0.049	0.112^*^
ACSQEbr	0.054	0.076	−0.037	−0.111	−0.037	−0.055	0.041	−0.005	0.005	0.246^***^	−0.046	0.181^***^	0.130^*^
ACSQEss	0.097	−0.022	−0.090	−0.110	−0.056	−0.010	0.061	−0.066	−0.023	0.102	−0.014	0.093	−0.058

Correlations are significant at the levels

* p < 0.05;

** p < 0.01;

*** *p < 0.001 (bilateral). ACSQ, Approach to Coping in Sport Questionnaire; ACSQUem, Use of emotional calm; ACSQUcr, Use of cognitive restructuring; ACSQUmw, Use of mental withdrawal; ACSQUbr, Use of behavioral risk; ACSQUss, Use of seeking social support; ACSQEem, Efficacy of emotional calm; ACSQEcr, Efficacy of cognitive restructuring; ACSQEmw, Efficacy of mental withdrawal; ACSQEbr, Efficacy of behavioral risk; ACSQEss, Efficacy of seeking social support; STAI-T, Trait Anxiety Inventory; DASS, Depression, Anxiety, and Stress Scales; DASSd, Depression; DASSa, Anxiety; DASSs, Stress; POMS, Profile of Mood States; POMSt, Tension; POMSd, Depression; POMSa, Anger; POMSv, Vigor; POMSf, Fatigue; FMPS, Multidimensional Perfectionism Scale; FMPSA, Adaptative; FMPSD, Dysfunctional*.

There are significant, negative correlations between the emotional states perceived as negatives (anxiety, depression, stress, and fatigue) and the use of coping strategies in athletes, such as cognitive restructuring and emotional calming.

Table 1 shows a high number of correlations in all the psychological variables considered, especially in the variables of personality traits (anxiety and perfectionism), mood states (POMS), and mental health (DASS-21), and to a lesser extent in sleep variables (except for sleep quality).

The magnitude of these correlations is moderate (medium or relatively low), ranging from significant values of *rho* = 0.119 (*p* < 0.05) to values of *rho* = 0.413 (*p* < 0.001). In most of the significant correlations found, the probabilities are *p* < 0.001.

The greatest number of correlations found when considering coping strategies (use and efficacy) was realized when crossing them with the STAI-T, the three factors of the DASS-21, mood states, and, to a lesser extent, with those related to the quality of sleep.

Table 2 shows the correlations, through the Spearman correlation coefficient, between the sleep questionnaire STAI-T, DASS-21, POMS, and FMPS questionnaires. Sleep quality correlates significantly with most of the measured variables. Sleep hours correlates significantly with some variables, all correlations being negative.

**Table 2:** Spearman correlation coefficient between the Sport Sleep Questionnaire and STAI-T, DASS-21, POMS, and FMPS questionnaires.

	**SH**	**STAI**	**DASSd**	**DASSa**	**DASSs**	**POMSt**	**POMSd**	**POMSa**	**POMSv**	**POMSf**	**FMPSA**	**FMPSD**
SQ	0.476^***^	−0.348^***^	−0.272^***^	−0.370^***^	−0.374^***^	−0.302^***^	−0.350^***^	−0.295^***^	0.267^***^	−0.311^***^	0.077	−0.161^**^
SH		−0.152^**^	−0.101	−0.261^***^	−0.260^***^	−0.212^***^	−0.168^**^	−0.161^**^	0.076	−0.146^**^	−0.001	−0.044

Correlations are significant at the levels

** p < 0.01 and

*** *p < 0.001 (bilateral). Sport Sleep Questionnaire; SQ, Sleep quality; SH, Sleep hours; DASS, Depression, Anxiety, and Stress Scales; DASSd, Depression; DASSa, Anxiety; DASSs, Stress; POMS, Profile of Mood States; POMSt, Tension; POMSd, Depression; POMSa, Anger; POMSv, Vigor; POMSf, Fatigue; FMPS, Multidimensional Perfectionism Scale; FMPSA, Adaptative; FMPSD, Dysfunctional*.

With the exception of the correlations found between the sleep measures (quality and hours, *rho* = −476; *p* < 0.001), the correlational values found range from *rho* = −0.134 (*p* < 0.05) to *rho* = 0.374 (*p* < 0.001). Therefore, most of the significant correlations found show a moderate or relatively small magnitude, observing how most correlations are obtained at a significance level of *p* < 0.001.

Table 3 shows the correlations, through the Spearman correlation coefficient, between the FMPS factors and the POMS, DASS-21, and STAI-T questionnaires. The FMPSD correlated significantly with all the measures evaluated. FMPSA only correlated significantly and positively with POMSt and POMSv. As in previous tables, the correlations found are of medium or relatively low magnitude, ranging from values of *rho* = 0.137 (*p* < 0.05) to the highest values found with the STAI of *rho* = 0.516 (*p* < 0.001).

**Table 3:** Pearson correlation coefficient between the FMPS, POMS, and DASS-21 questionnaires.

	**POMSt**	**POMSd**	**POMSa**	**POMSv**	**POMSf**	**DASSd**	**DASSa**	**DASSs**	**STAI**
**FMPSA**	0.137^*^	−0.076	0.108	0.256^***^	−0.056	−0.054	0.021	0.105	−0.004
**FMPSD**	0.294^***^	0.283^***^	0.270^***^	−0.126^*^	0.304^***^	0.323^***^	0.322^***^	0.334^***^	0.516^***^

Correlations are significant at the levels

* p < 0.05 and

*** *p < 0.001 (bilateral). STAI-State, Trait Anxiety Inventory; DASS, Depression, Anxiety, and Stress Scales; DASSd, Depression; DASSa, Anxiety; DASSs, Stress; POMS, Profile of Mood States; POMSt, Tension; POMSd, Depression; POMSa, Anger; POMSv, Vigor; POMSf, Fatigue; FMPS, Multidimensional Perfectionism Scale; FMPSA, Adaptative; FMPSD, Dysfunctional*.

Table 4 presents the descriptive statistics and Kruskal–Wallis test for the STAI-T, POMS, DASS-21, and FMPS questionnaires, in relation to the variable sex. A *post-hoc* analysis has been performed using the Mann–Whitney *U*-test. Statistically significant differences were found in STAI-T (*p* < 0.001) and some factors of the POMS in relation to sex (POMSv, *p* < 0.009; POMSf, *p* < 0.05). DASS-21 scores reveal statistically significant differences in two of the three factors (DASS, *p* < 0.004; DASSs, *p* < 0.000). These results show how the sample of female athletes obtained the highest scores among all the variables considered, except for the POMS-VI, FMPSA, and FMPSD, where male athletes obtained the highest scores. From all these variables, only significant differences are shown with a *p* < 0.001 in the STAI, DASS-21 EST, and STAI-Total; with a *p* < 0.01 in DASS-21-AS and POMS-VI; and with a *p* < 0.05 in POMS-FA.

**Table 4:** Descriptive statistics and Mann–Whitney *U*-test of the STAI-T, POMS, DASS-21, and FMPS, for the sex variable.

	**TOTAL X (SD) (*N* = 310)**	**Male X (SD) (*n* = 169)**	**Female X (SD) (*n* = 141)**	***Z***	***Sig (p)***
**STAI-T Factor**					
STAI R(0–60)	20.80 (9.57)	19.21 (9.00)	22.68 (9.92)	−3.212	0.001^***^
**POMS Factors**					
POMSt R(0–24)	4.39 (4.52)	3.85 (3.97)	5.04 (5.03)	−1.860	0.063
POMSd R(0–20)	3.25 (3.24)	3.22 (3.06)	3.30 (3.46)	−0.259	0.796
POMSa R(0–32)	8.22 (5.23)	7.97 (4.76)	8.52 (5.75)	−0.320	0.749
POMSv R(0–20)	11.00 (3.86)	11.50 (3.73)	10.42 (3.95)	−2.612	0.009^**^
POMSf R(0–20)	4.40 (3.83)	3.94 (3.73)	4.95 (3.90)	−2.500	0.012^*^
**DASS-21factors** ^*^R(0–21)					
DASSd	4.30 (3.55)	4.00 (3.27)	4.65 (3.83)	−1.229	0.219
DASSa	2.28 (2.77)	1.76 (2.45)	2.89 (2.99)	−2.877	0.004^**^
DASSs	4.89 (4.28)	4.20 (3.84)	5.72 (4.62)	−3.938	0.000^***^
**FMPS factors**					
FMPSA R(7–35)	46.80 (8.78)	46.99 (8.77)	46.57 (8.82)	−0.112	0.911
FMPSD R(6–30)	54.04 (14.23)	54.64 (14.48)	53.32 (13.94)	−0.783	0.434

X (SD) = Average (Standard Deviation); R = Rank;

* R = Full Scale Rank. STAI-T, Trait Anxiety Inventory; DASS, Depression, Anxiety, and Stress Scales; DASSd, Depression; DASSa, Anxiety; DASSs, Stress; POMS, Profile of Mood States; POMSt, Tension; POMSd, Depression; POMSa, Anger; POMSv, Vigor; POMSf, Fatigue; FMPS, Multidimensional Perfectionism Scale; FMPSA, Adaptative; FMPSD, Dysfunctional. Significant levels at

* p < 0.05;

** p < 0.01;

*** *p < 0.001 (bilateral)*.

Table 5 shows the descriptive statistics and Kruskal–Wallis test for the ACSQ-Use/Efficacy questionnaire and the two items subtracted from the Sports Sleep Questionnaire, in relation to the variable sex. The results show no significant differences between male and female participants in their perception coping strategies, hours of sleep, and perception of quality of sleep.

**Table 5:** Descriptive statistics and Mann–Whitney *U*-test of the ACSQ and Sport Sleep Questionnaires, for the sex variable.

	**TOTAL X (SD) (*N* = 310)**	**Male X (SD) (*n* = 169)**	**Female X (SD) (*n* = 141)**	**MW-*U***	***Z***	***Sig (p)***
**ACSQ-Use/Efficacy**						
ACSQUec R(7–35)	24.18 (5.21)	23.73 (5.62)	24.73 (4.66)	10,637.00	−1.645	0.100
ACSQUcr R(6–30)	22.00 (5.06)	21.63 (5.21)	22.43 (4.86)	11,003.50	−1.179	0.239
ACSQUmw R(6–30)	12.44 (5.06)	12.25 (5.16)	12.65 (4.94)	11,301.00	−0.800	0.424
ACSQUbr R(4–20)	9.16 (3.96)	9.32 (3.71)	8.98 (4.25)	11,164.00	−0.977	0.329
ACSQUss (5–25)	10.50 (4.94)	10.14 (4.57)	10.93 (5.34)	11,138.50	−1.009	0.313
ACSQEec R(7–35)	24.96 (6.074)	24.71 (6.27)	25.27 (5.84)	11,409.50	−0.661	0.509
ACSQEcr R(6–30)	21.77 (5.67)	21.52 (5.62)	22.06 (5.74)	11,144.00	−1.000	0.318
ACSQEmw R(6–30)	12.26 (5.65)	12.32 (5.94)	12.20 (5.32)	11,716.50	−0.271	0.787
ACSQEbr R(4–20)	10.08 (4.38)	10.23 (4.28)	9.91 (4.50)	11,316.00	−0.782	0.434
ACSQEss R(5–25)	13.38 (6.17)	12.91 (5.85)	13.94 (6.50)	10,832.50	−1.398	0.162
**Sleep**						
SQ R(1–5)	350 (1.21)	3.58 (1.21)	3.41 (1.21)	10,912.00	−1.331	0.183
SH	7.68 (1.58)	7.75 (1.56)	7.61 (1.61)	11,278.50	−0.760	0.447

X (DS) = Average (Standard Deviation); R = Rank; *R = Full Scale Rank. ACSQ, Approach to Coping in Sport Questionnaire; ACSQUem, Use of emotional calm; ACSQUcr, Use of cognitive restructuring; ACSQUmw, Use of Mental withdrawal; ACSQUbr, Use of behavioral risk; ACSQUss, Use of seeking social support; ACSQEem, Efficacy of emotional calm; ACSQEcr, Efficacy of cognitive restructuring; ACSQEmw, Efficacy of mental withdrawal; ACSQEbr, Efficacy of behavioral risk; ACSQEss, Efficacy of seeking social support. Sport Sleep Questionnaire; SQ, Sleep Quality; SH, Sleep hours. Significant levels at *p < 0.05; **p < 0.01; ***p < 0.001 (bilateral).

Table 6 presents the descriptive statistics and analysis of the mean difference between the different sports modalities considering the responses of the STAI-T, POMS, DASS21, and the FMPS, applying the Kruskal–Wallis test for the multiple independent samples. *Post-hoc* analysis have been performed using the mean difference analysis for two independent Mann–Whitney *U*-samples. The mean scores obtained on STAI-T, POMS, and DASS21 do not show significant differences in relation to sports disciplines (*p* < 0.05). The relationship between FMPSD and MA presents a remarkable average score compared to the rest of sports. The results show statistically significant relationships in FMPS.

**Table 6:** Descriptive statistics obtained from the FMPS and STAI questionnaires and Kruskal–Wallis test with *post-hoc* analysis with Mann–Whitney *U*-test (MW-U) carried out on the variable sport practiced.

		**X (SD)**	**χ^2^**	**Sig**.	**MW-U**	**Sig**.
**STAI**	Athletics	18.89 (8.62)		0.142		
	Football	20.75 (9.17)	12.202			
	Rugby	23.83 (11.60)				
	Basketball	22.24 (11.41)				
	Cycling/triathlon	19.00 (5.80)				
	Martial arts	21.03 (8.94)				
	Climbing	12.82 (7.25)				
	Swimming/water polo	23.17 (8.54)				
	Other sports	20.65 (9.53)				
**FMPSA**	Athletics	46.42 (8.11)	24.681	0.001^***^	415.000	0.013^*^
	Football	43.84 (10.11)			404.500	0.000^***^
	Rugby	43.88 (7.76)			1497.500	0.006^**^
	Basketball	46.59 (7.33)			192.500	0.000^***^
	Cycling/triathlon	44.00 (15.22)			744.500	0.007^**^
	Martial arts	50.60 (7.65)			321.000	0.012^*^
	Climbing	44.27 (6.78)			85.500	0.006^**^
	Swimming/water polo	45.00 (7.69)			333.000	0.045^*^
	Other sports	48.44 (8.33)				
**FMPSD**	Athletics	51.61 (12.52)	27.691	0.002^**^	94.500	0.009^**^
	Football	48.61 (10.23)			536.000	0.021^*^
	Rugby	51.50 (12.13)			1360.500	0.001^***^
	Basketball	52.97 (11.10)			64.000	0.016^*^
	Cycling/triathlon	45.33 (16.14)			67.500	0.005^**^
	Martial arts	55.91 (15.64)			129.500	0.049^*^
	Climbing	41.91 (7.66)			338.500	0.020^*^
	Swimming/water polo	58.33 (16.45)			79.000	0.003^**^
	Other sports	58.10 (15.20)			12.000	0.035^*^
					179.500	0.000^***^

X (DS) = Average (Standard Deviation); R = Rank; FMPS, Multidimensional Perfectionism Scale; FMPSA, Adaptative; FMPSD, Dysfunctional; STAI-State, Trait Anxiety Inventory. Level of significance:

* p < 0.05;

** p < 0.01;

*** *p < 0.001*.

It can be seen how the highest scores on the STAI are obtained by Rugby players followed by basketball. In POMS-TE, DE, and CO, the highest scores have been obtained by soccer, basketball, and rugby players. At Vigor, the highest scores are obtained by swimmers/water polo players and MA wrestlers. The highest levels of Fatigue are observed in swimmers and fighters of MA.

Regarding the values of the DASS21, the highest scores in DE are obtained in MA fighters and basketball players. At ANS, the highest scores are obtained in swimming and basketball. In EST, the highest scores are obtained from swimmers, footballers, and cyclists/triathletes. The most adaptive perfectionist athletes are MA wrestlers, and the most maladaptive are MA swimmers/water polo players and wrestlers.

Despite the scores shown, only significant differences are shown by applying the Kruskal–Wallis test in Adaptive perfectionism (*p* < 0.001) and maladaptive perfectionism (*p* < 0.01). In both cases, there is a very high number of differences between groups of athletes, and many of them of *p* < 0.01. or *p* < 0.001.

Similar to the results obtained in Table 6, the application of *post-hoc* tests on the six variables using the Mann–Whitney *U*-test shows a very high number of significant differences between sports, finding in many of them a great variability in scores between sports (*p* < 0.01 or <0.001).

The descriptive statistics analysis and the Kruskal–Wallis test for the sport variable in relation to the results obtained in the ACSQ-U questionnaire are shown in Table 7. *Post-hoc* analysis have been performed using the mean difference analysis for two independent Mann–Whitney *U*-samples. Statistically significant differences were observed in emotional calming (*p* < 0.01) and seeking social support (*p* < 0.04).

**Table 7:** Descriptive statistics and Kruskal–Wallis test with *post-hoc* analysis with Mann–Whitney *U*-test carried out on the sport variable in relation to the scores obtained in the ACSQ-U.

**Test/Sport**		**X (SD)**	***χ*^2^**	**Sig**.	**Mann–Whitney**	***Z***	**Sig**.
**ACSQUec**	Athletics	24.67 (5.060)	19.96	0.01^**^			
	Football	22.73 (5.11)			448.50	−2.09	0.036^*^
	Rugby	24.38 (4.48)			409.50	−3.56	0.000^***^
	Basketball	22.41 (4.62)			1653.50	−2.06	0.039^*^
	Cycling/triathlon	22.92 (6.44)			269.00	−2.33	0.019^*^
	Martial arts	26.89 (4.77)			243.00	−3.58	0.000^***^
	Climbing	23.18 (5.70)			964.50	−2.50	0.012^*^
	Swimming/water polo	24.00 (4.60)			112.50	−2.06	0.039^*^
	Other sports	24.85 (5.48)			1277.00	−2.10	0.036^*^
**ACSQUcr**	Athletics	22.75 (4.61)	7.15	0.52			
	Football	21.07 (4.83)					
	Rugby	22.71 (3.77)					
	Basketball	20.76 (5.90)					
	Cycling/triathlon	21.83 (6.39)					
	Martial arts	23.69 (3.84)					
	Climbing	22.18 (6.24)					
	Swimming/water polo	21.33 (4.84)					
	Other sports	21.96 (5.41)					
**ACSQUmw**	Athletics	10.72 (4.60)	9.19	0.32			
	Football	11.82 (4.42)					
	Rugby	13.42 (4.42)					
	Basketball	13.48 (5.63)					
	Cycling/triathlon	13.00 (3.27)					
	Martial arts	11.71 (3.98)					
	Climbing	12.09 (5.31)					
	Swimming/water polo	12.67 (5.50)					
	Other sports	13.11 (5.89)					
**ACSQUbr**	Athletics	9.03 (3.78)	12.39	0.13			
	Football	8.80 (3.56)					
	Rugby	8.42 (3.79)					
	Basketball	8.24 (3.48)					
	Cycling/triathlon	8.58 (4.54)					
	Martial arts	11.06 (3.75)					
	Climbing	8.73 (3.28)					
	Swimming/water polo	10.17 (3.54)					
	Other sports	9.31 (4.52)					
**ACSQUss**	Athletics	11.83 (5.54)	16.09	0.041^*^			
	Football	9.05 (4.00)			558.000	−2.274	0.023^*^
	Rugby	10.13 (4.079)			373.500	−1.968	0.049^*^
	Basketball	8.93 (3.34)			86.000	−3.573	0.000^***^
	Cycling/triathlon	14.33 (4.53)			78.500	−2.206	0.027^*^
	Martial arts	11.29 (4.16)			55.000	−3.424	0.001^***^
	Climbing	10.27 (5.85)			12.500	−2.212	0.027^*^
	Swimming/water polo	9.17 (4.35)			354.000	−2.181	0.029^*^
	Other sports	10.86 (5.42)					

X (SD) = Average (Standard Deviation); ACSQ, Approach to Coping in Sport Questionnaire; ACSQUec, Use of emotional calm; ACSQUcr, Use of cognitive restructuring; ACSQUmw, Use of mental withdrawal; ACSQUbr, Use of behavioral risk; ACSQUss, Use of seeking social support. Significant levels at

* p < 0.05;

** p < 0.01;

*** *p < 0.001 (bilateral)*.

In the descriptive table, it can be observed that MA athletes got the highest scores in ACSQ_U_CE, ACSQ_U_RC, ACSQ_U_CR, ACSQ_E_CE, and ACSQ_E_RC. In the ACSQ_U_RM variable, basketball players, followed closely by rugby players, are the ones that got the highest scores. The variable ACSQ_U_BAS highlights the highest scores for cycling and triathlon. In the variable ACSQ_E_RM, the highest scores for rugby players stand out. In the variable ACSQ_E_CR, the highest scores of the swimmers' group stand out.

The descriptive statistics analysis and the Kruskal–Wallis test for the sport variable in relation to the results obtained in the ACSQ-E questionnaire are shown in Table 8. *Post-hoc* analysis have been performed using the mean difference analysis for two independent Mann–Whitney *U*-samples. Statistically significant differences were observed in emotional calming (*p* < 0.04), mental withdrawal (*p* < 0.01), and seeking social support (*p* < 0.00). The martial arts sport discipline presents slightly higher average scores than the other sports disciplines in most factors.

**Table 8:** Descriptive statistics and Kruskal–Wallis test with *post-hoc* analysis with Mann–Whitney *U*-test carried out on the sport variable in relation to the scores obtained in the ACSQ-E.

**Test/Sport**		**X (SD)**	**χ^2^**	**Sig**.	**Mann–Whitney**	***Z***	**Sig**.
**ACSQEec**	Athletics	24.97 (6.98)	17.072	0.049*			
	Football	23.16 (7.06)			514.500	−2.528	0.011*
	Rugby	25.96 (4.46)			217.000	−2.350	0.019*
	Basketball	22.48 (5.61)			257.000	−3.387	0.001***
	Cycling/triathlon	23.33 (7.39)			76.000	−2.537	0.011*
	Martial arts	27.09 (4.55)			942.000	−2.639	0.008**
	Climbing	27.00 (6.84)					
	Swimming/water polo	25.33 (4.80)					
	Other sports	25.75 (5.87)					
**ACSQEcr**	Athletics	22.67 (5.17)	13.202	0.105			
	Football	19.73 (5.91)					
	Rugby	21.79 (4.94)					
	Basketball	20.07 (6.19)					
	Cycling/triathlon	23.17 (5.70)					
	Martial arts	23.80 (4.50)					
	Climbing	22.18 (6.40)					
	Swimming/water polo	22.50 (4.03)					
	Other sports	21.95 (5.99)					
**ACSQEmw**	Athletics	11.83 (5.95)	19.642	0.012*	260.500	−2.607	0.009**
	Football	9.89 (4.30)			224.000	−3.933	0.000***
	Rugby	15.96 (6.04)			416.000	−2.527	0.012*
	Basketball	12.72 (5.16)			138.500	−2.532	0.011*
	Cycling/triathlon	13.67 (5.77)			555.500	−2.138	0.033*
	Martial arts	12.31 (5.55)			1637.000	−2.153	0.031*
	Climbing	13.27 (6.84)			238.000	−1.972	0.049*
	Swimming/water polo	12.33 (7.94)			262.500	−2.439	0.015*
	Other sports	12.21 (5.74)			738.500	−2.727	0.006**
**ACSQEbr**	Athletics	10.17 (4.53)	9,376	0.312			
	Football	9.32 (3.57)					
	Rugby	10.04 (4.41)					
	Basketball	8.72 (4.43)					
	Cycling/triathlon	9.50 (4.54)					
	Martial arts	10.86 (4.42)					
	Climbing	9.91 (5.06)					
	Swimming/water polo	12.50 (3.01)					
	Other sports	10.58 (4.73)					
**ACSQEss**	Athletics	16.81 (6.48)	20,276	0.009**			
	Football	24.97 (6.98)					
	Rugby	23.16 (7.06)			453,500	−3.285	0.001***
	Basketball	25.96 (4.46)			265,500	−2.521	0.012*
	Cycling/triathlon	22.48 (5.61)			267,000	−3.373	0.001***
	Martial arts	23.33 (7.39)			85,000	−1.988	0.047*
	Climbing	27.09 (4.55)			80,000	−2.701	0.007**
	Swimming/water polo	27.00 (6.84)					
	Other sports	25.33 (4.80)					

*X (SD) = Average (Standard Deviation); ACSQ, Approach to Coping in Sport Questionnaire; ACSQEec, Efficacy of emotional calm; ACSQEcr, Efficacy of cognitive restructuring; ACSQEmw, Efficacy of mental withdrawal; ACSQEbr, Efficacy of behavioral risk; ACSQEss, Efficacy of seeking social support*.

Applying the Kruskal–Wallis test, statistically significant results are shown with a *p* < 0.01 in ACSQ_E_BAS and ACSQ_U_CE, and with a *p* < 0.05 in ACSQ_E_RM, ACSQ_U_CE, ACSQ_U_BAS, and ACSQ_E_CE. The application of the *post-hoc* test in the six variables using the Mann–Whitney *U*-test shows a high number of significant differences between sports, finding major score differences among sports in many of them (*p* < 0.01 or <0.001).

**Figure 1:**
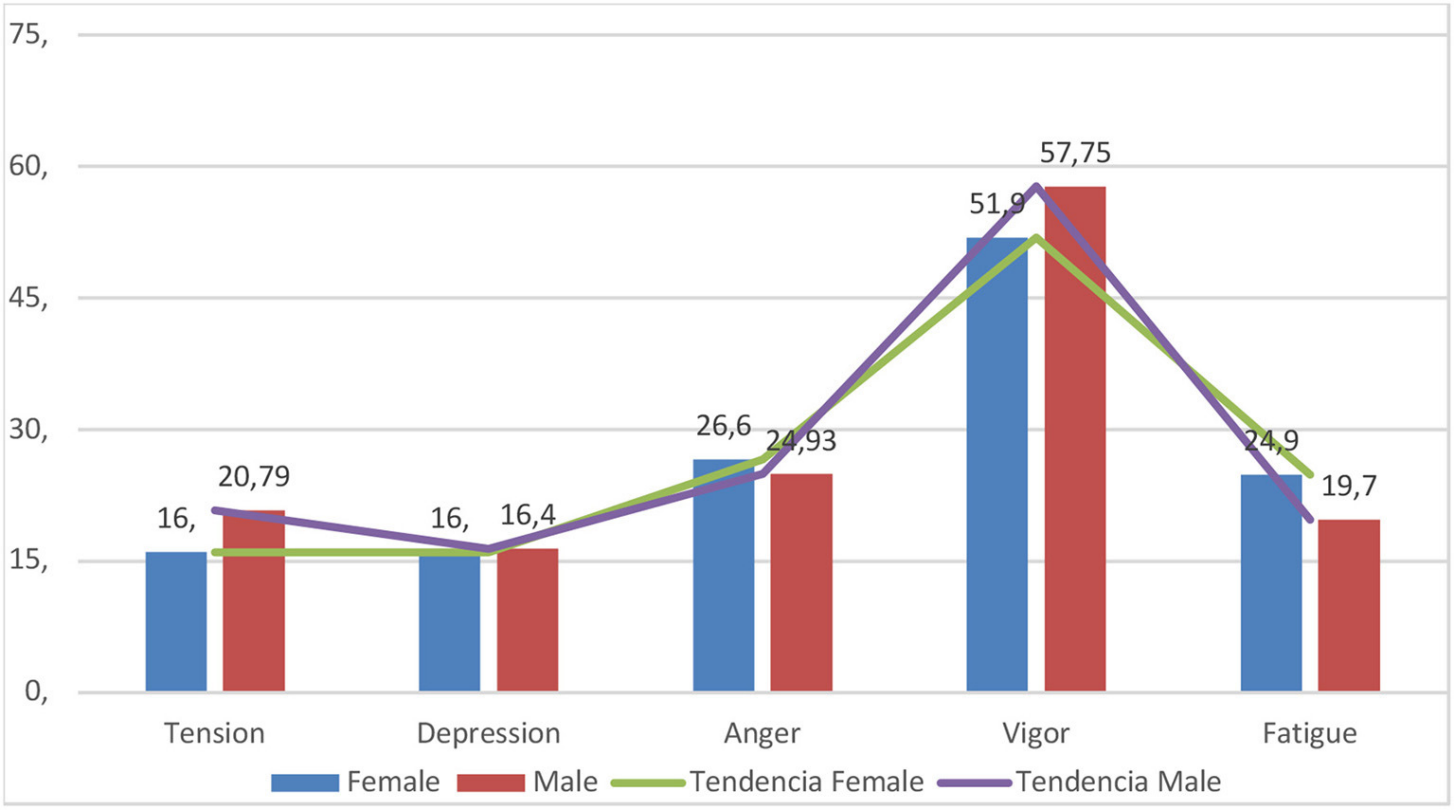
Female and male profile in the POMS Scales.

Figure 1 shows the profile corresponding to the average results of the POMS in the sample. As seen, both male and female athletes present an ideal mood profile, in line with the iceberg profile in which the vigor factor is at levels higher than the other factors.

Anxiety and depression scores collected through various instruments (STAI, DASS-21, and POMS) correlate significantly with each other, which indicates the reliability of the results obtained.

## Discussion

Due to the unexpected force of the COVID-19 outbreak's social impact, researchers from around the world and from a wide variety of disciplines responded promptly, applying accessible and previously applied models most of the time. In the case of sports psychology, the first model used was the well-known Model of Sports Injuries [(43), MGLD: (9)] due to the similarity in the interruption of activity and the inherent uncertainty of the return to normal sports conditions, especially in long-term injuries. In the latest formulations, this model had added other variables such as self-efficacy (44) and environmental factors to the primary concepts of anxiety and stress. Likewise, unique confinement experiences and their associated psychological aspects were studied, although it did not offer conclusive results (17, 18).

To mitigate this bias, the protocol designed for this particular study included several other variables, from coping strategies, anxiety, perfectionism, to behavioral ones, such as the perception of quality and quantity of sleep. In order to provide more control to the cross-sectional set of data, we collected it at least 15 days after the confinement began in each of the represented countries. At that time, there was no official plan to return to regular training sessions and hardly any expectation of resuming competitions at any sports level. In contrast, there was a continuous trickle of announcements informing cancellations and official postponements of major events, as was the worldwide emblematic case of the Tokyo Olympic Games (postponed to 2021).

Overall, the results of the present study show significant, negative correlations between the use of coping strategies in athletes, mainly on cognitive restructuring and emotional calming, and the emotional states commonly labeled as negative, such as depression, stress, anxiety, and fatigue. Although minor differences were found between males and females, the latter have shown higher scores in most psychological variables studied. The study of differences based on the sex variable is a relevant line of research where several studies have recently been carried out in this regard (45). However, it will be necessary to carry out specific investigations in this field of study. When it comes to sport disciplines, there are no significant differences among them except in the case of martial arts and the variable perfectionism, both subscales (adaptive perfectionism and maladaptive perfectionism). Implications of this last piece of data are discussed later in this section.

The results collected in this study have questioned in some way our expectations of the potential psychological impact on high-performing athletes, even when analyzed cross-culturally. According to our results, we can first observe that the main “clinical” values (a specific aspect of self-perception of mood), such as perceived stress, anxiety, or “depressive” symptoms, are relatively low. Within this triad, perception of stress was the highest value recorded, but without crossing the pathological range in any case. According to multiple previous studies, especially those related to anxiety and depression, a slight but significant difference can be observed toward higher values in female athletes than in male ones. The implication of this fact regarding performance is deeply debated by other authors and previous studies (46, 47).

In congruence with our results, Clemente-Suarez et al. (48) recently found little to no impact of confinement on the levels of anxiety of Olympic and Paralympic athletes. Authors attributed these phenomena to the larger experience of high-performance athletes in coping with competition-related anxiety and the higher cognitive resources professional athletes have. Similar results were observed in professional chess players by Fuentes-García et al. (49) despite the decrease of physical activity, which suggests that the cognitive resources of athletes potentially mitigate the negative effects of confinement. However, the same studies previously mentioned showed contradictory results in terms of academic levels and subjective perception of the confinement situation. On the one hand, chess players with university studies showed greater concern about the COVID-19 pandemic (49), while Olympic athletes with higher education showed more dissatisfaction with the confinement measures as a result of the COVID-19 pandemic (48).

In the second level of analysis related to mood states, it is interesting to observe that, using classic and contrasting measures with other studies (50) and, in the same line as findings discussed in the previous paragraph, the profiles match almost perfectly with the so-called “Iceberg profile” associated with high performance (38, 51, 52), despite a slight deviation corresponding to sex, a well-researched phenomenon reported in normal situations (53). It is highlighted that Vigor scores are the highest, while Depression scores are far below, clearly indicating that athletes have not experienced a decrease in their perception of energy in this situation of confinement (54).

Indeed, the results obtained regarding the athletes' perfectionism, as was indicated (21), supported all the results in the emotional spectrum. Nevertheless, the mean values of this trait never reached a considerable level among the athletes studied. However, inquiring into their possible connections, the values of the “good” and healthy perfectionism correlated positively with the mood state factors of Tension and Vigor. In a genuine sense, this fact adds no operative knowledge to our study, but there is no doubt that it enhances the facts found related to some authors advocating for more studies focused on the elite athletes' personality profiles, perhaps just one more brick on the wall. In another study carried out during confinement with a sample of Russian and Bulgarian university athletes, high values in adaptive perfectionism have been found, also significantly correlating with positive moods and adaptive coping strategies (55).

Complementarily and as expected theoretically, the “bad” perfectionism correlated negatively and significantly with Vigor and Tension POMS' factors, and positively with the negative ones, such as depression. This very same fact appears in all the aforementioned “clinical” factors considered (Depression, Anxiety, and Stress), and in trait anxiety personality scores. Overall, we must consider that lower values in our sample indicate that their skills or abilities were robust enough to overcome the constellation of black clouds surrounding this abnormal situation for elite athletes.

High-performance athletes follow strict training schedules to minimize the risk of making mistakes in competition; therefore, they train to perfect their technique and become meticulous with the “shape” of their execution. Although a certain degree of perfectionism is acceptable and expected in high-performance athletes, our results show interesting differences in perfectionism among sports disciplines. Martial arts (MA) is the sports discipline with the highest perfectionism (both adaptive and dysfunctional), followed by swimming. In practical terms, a single mistake in martial arts can lead to a knockout, which means losing the competition, and consequential physical risk (e.g., injury, death). Even if perfectionism may help reduce the chances of making mistakes, it also entails low tolerance for error, which, combined with inadequate stress-management mechanisms developed to avoid failure, could negatively impact performance. We are aware that this fact is beyond the scope of this paper; however, we find this evidence is worth mentioning.

Regarding the concept of perfectionism, the differences found according to sex are in line with the differences found in the variables anxiety and mood states already presented by other authors (56, 57). It is worth noting the importance of developing specific psychological tests to analyze relevant variables such as “Sports age category by sex” and “Sports age category” and may include other physical variables such as body mass index (BMI) in the study if needed, in line with previous research that refers to the existence of correlations between physical and psychological variables (44).

Therefore, we can assume that no indicator of psychological dysfunction has been observed in the sample of top-level athletes studied, supporting the findings of other confinement situations, in which only some signs of slowing cognitive processing appeared (58, 59) along with displacement of sleep phase, a typical syndrome of workers on night shifts, or the absence of perceived *zeitgebers* (17, 60).

Reinforcing this argument, another significant fact is related to the main behavioral indicator of this study: hours of sleep and the perceived quality of sleep. Sleep has been positively reported, meaning that athletes in the sample expressed a good quality of sleep and proper amounts of hours of sleep, allowing sleep to be perceived as restorative. However, we cannot conclude that alteration of circadian rhythms has not taken place since it remains plausible that athletes suffered changes in their bedtime routine and wake-up schedules because of the lack of objective measures regarding possible phase shifts.

In general terms, it can be observed that emotional state scores are low, interpreted as pleasant moods. Perhaps the “modulating” variable of these values is the use of coping strategies assessed with the ACSQ questionnaire, minimizing the negative psychological impact that the restrictive measures of this pandemic were expected to cause. Athletes mainly used coping strategies based on “emotional calming” and “search for social support,” both perceived as effective strategies for emotional regulation (61). It deserves to be noted that these coping strategies are present in a cross-cultural sample of athletes, in comparison with the “textbook” behavioral approaches (62). Supporting this fact, it is also confirmed that the use of coping strategies correlates negatively and significantly with emotional states perceived as negative (anxiety, depression, stress) regardless of the low values of the latter.

Despite what is stated in the previous paragraph, and from the authors' point of view, these results, when critically analyzed, may “add fuel” to the already classic discussion between supporting the existence of a trait of hardiness (63), grit (64), or mental toughness (65), and supporting the relevance of training in coping strategies during the career of high-performance athletes. It is a very important discussion between modifiable and trainable variables or traits that can be otherwise detected and promoted (66, 67). From our data, a point appears in favor of the first of the assumptions, since the trait anxiety values have been remarkably low in the entire sample studied, with no screening being carried out before the inclusion of the practitioners. Certainly, this issue is more than susceptible to further research.

However, the authors acknowledge that even taking into account one of the most important limitations of the study, which we were not able to carry out because of technical and time constraints, the use of a mixed methodology (68) would allow for a more thorough analysis of the similarities of this confinement situation to some aspects of the psychological experiences of injured athletes. Perhaps the most relevant of these characteristics is the analysis of the psychological phases considered temporarily through which athletes with long-term injuries must face, especially those in which the time of return to practice is quite uncertain than more predictable forms of injury [very similar to those that appear in the well-known “psychological pain,” for example, (69, 70)].

Although clearly anecdotal, we have a supporting statement by a well-known, high-level world athlete, Rafael Nadal, who, 3 weeks after confinement was declared in Spain, announced through social media that he has decided to change his attitude toward the complicated situation caused by the coronavirus pandemic, confined as he is in Mallorca. “Good morning everyone, here we are. Difficult moments, however less time remains. As of today, I have tried to make a change, to be positive, and count the days that pass, because there is less time left. Greetings to you all and lots of encouragement, Vamos!” said the tennis player (71).

This line of thought leads us to agree with the reality that many of the previously studied confinement situations (58, 72) had very strict and elaborate work objectives, with previously established schedules that almost fully covered the subjects' time. Given this is definitely not the case (e.g., when athletes still do not have clear and reliable training plans to return to the practices and competitions), we must not forget the importance of self-established objectives (the data of the coping strategies of autonomy in decisions supports this suggestion), as it was highlighted by Glyn Roberts when arguing in favor of goal setting techniques in performance athletes (73). Speaking of which, this strategy has recently been confirmed to be effective in treating the psychological impact of medium- and long-term sports injuries (31).

Nevertheless, a specific conceptual model proposal has recently appeared [“Coronavirus Experience,” CE, (74)] that is based on their previous Scheme of change for sport psychology practice model (75) that is not far from the model of response to sports injuries. CE is defined as an unpredictable, longitudinal, and multifaceted change event that consists of three phases: phase A, characterized by instability and confusion, emotional response, and appraisal; phase B, defined by coping and regression; and phase C, in which instability can increase or decrease depending on the previous sporting trajectory.

Continuing with the analogy of sports injuries, when most severe situations of confinement come to an end, it is likely that some athletes may develop post-traumatic stress symptoms (76), since, as in sports injuries, concerns over the injury process often arise, such as re-injury, when athletes resume training (29). Data analysis leads us to consider the possibility that fear of contagion or worry regarding another alarm state that involves confinement may be present in athletes once “normal” life is reestablished.

Therefore, the data collected does not corroborate the analogy with which this study began: to compare the possible psychological impact of the confinement due to the COVID-19 pandemic with the sports injury process. It is possible to establish that the athletes' experience of confinement did not suppose a significant, negative experience since they could compare their situation with the rest of the athletes from their respective sports, who were also confined, as proposed by the Social Comparison Theory (77). On the other hand, another possible explanation is that just as it happens when an athlete experiences an injury, confinement might have brought an opportunity to perceive a certain degree of control of the situation, including the implementation of strategies to improve psychological well-being that might promote the appearance of the phenomenon known as post-traumatic growth, consequently avoiding the impact of negative mood states on performance (78, 79).

Finally, it can be inferred from the results that sleep parameters, both objective and subjective, could constitute a light indicator to indirectly recognize the emotional state of athletes since it correlates negatively and significantly with emotional states perceived as negative (anxiety, depression, stress, fatigue, tension, and anger). As previously explained, sleep is a very sensitive factor to any sign of stress (80).

### Limitations of the Study

This study has a series of evident limitations that we wish to indicate:

1. To ensure the quality of the data collected, we worked with those performance centers of our various research networks that (i) agreed to give a quick and supervised response from their athletes and (ii) had a sports psychology department that allowed us to homogenize the results obtained. This undoubtedly reduced the accessible sample, but we believe that it improved its reliability instead.
2. The models used determined the psychological variables used in the evaluation, which, in light of the results obtained, will require a major revision in the follow-ups of the sample that will be carried out in the phase of return to sports practice.
3. This study provides a relatively narrow time window, which may or may not be applicable to other, perhaps longer, lockdown situations.
4. Lastly, classical statistics have been used to analyze the collected data, but the experience of our group indicates that the use of Bayesian analysis, for example, to analyze the data probabilistically, may offer a complementary perspective that is more oriented to the possible prediction, than to a posteriori explanation.

### Practical Applications

There seems to be a legitimate advantage in athletes who have seen their daily life altered and their future career affected, like the rest of mankind, but somehow managed to effectively cope with the psychological impact that was expected from an unprecedented crisis like this one. Since this research sample was accessed through the solidarity and joint efforts of an international network of psychology departments that are part of high-performance institutions, it could be assumed that most athletes in this sample have had some sort of contact with their respective psychology department throughout their professional careers. Also, the technical staff could have acted as an intermediator of psychological techniques and strategies that protect athletes from “giving up” in the face of challenges, even if some athletes in the sample might have not interacted directly with the sports psychology personnel in their institutions. If this interpretation proves accurate, high-school teachers and university professors, among other instructor figures, could be trained in coping strategies and crisis management to serve as role models for younger generations. Academic and institutional networks as the one that allowed this quick response to research obligations should be encouraged to provide high-speed examination of potential dangers.

Similar to the pathogenesis of COVID-19, psychological disorders are “invisible” to the general population until the symptomatology has reached a point where treatment is required. In the same way governments and institutions could have prevented the rate of dissemination of the coronavirus, psychological consequences of periods of crisis might be prevented as well. Protective factors against psychological disorders and promotion of well-being seem to have a strong relationship with the nature in which athletes have been trained to cope with stressful events. Our findings shed some light on the underlying psychological mechanisms that function as protective factors of negative mental states triggered by this emergency crisis. Ideally, preventive programs based on coping strategies and healthy habits could be transferred to the general public in organized sports and leisure activities. A more realistic approach could involve psychological training to those sectors of the community at risk of psychological dysfunction.

Also evident from the findings is the fact that, although the values of the “negative” psychological variables do not reach critical levels, their correlation with the athletes' sleep indicators is interesting and should not fall on deaf ears. The current facilities for collecting data in a mixed way (subjective and objective, such as by accelerometry) should be a standard monitoring practice—in the preventive line indicated in the previous point—within the protocols applied to high-performance athletes.

The results obtained in perfectionism follow the same line of other variables included in this research: there are differences in the sex variable. As already highlighted in previous research (56, 57), it is needed to develop future research examining certain variables of the athlete's personality and strictly sports-related, such as sports age category, sports category by sex, and category by weight, among other variables.

### Future Lines of Research

Future lines of research should consider a longitudinal analysis of how these psychological variables develop from the imposition of confinement to the reestablishment of normality.

The fact that only psychological variables have been considered from a strictly quantitative perspective is a limitation, mainly because in those studies developed where there is limited bibliographic review on the subject studied, the most advisable thing is to carry out a mixed analysis, both quantitative and qualitative. In novel research topics, it is relevant to collect data from the subjects through non-standardized or open-response techniques. Therefore, the development of mixed models (qualitative–quantitative) is recommended, although the quantitative information included in this study is relevant from a theoretical and empirical perspective.

## Data Availability Statement

The raw data supporting the conclusions of this article will be made available by the authors, without undue reservation.

## Ethics Statement

The studies involving human participants were reviewed and approved by University of Trás-os-Montes e Alto Douro (UTAD, Portugal) Ethical Committee, code 23/DOC20/CE/UTAD (27/06/2018). The ethics committee waived the requirement of written informed consent for participation.

## Author Contributions

AG-M and AO designed the study as a whole. AO designed the questionnaires' protocol and FL adapted them in the online version. FL, AG-M, and AN prepared the draft of the introduction, with all the co-authors contributing to the revision and final version. FL carried out the data collection. FV, VG-E, RR-B, and AN were in charge of the statistical analyzes. FL, AO, and AG-M prepared the first draft of the discussion, with all the co-authors contributing to the final version and revisions. All authors contributed to the article and approved the submitted version.

## Conflict of Interest

The authors declare that the research was conducted in the absence of any commercial or financial relationships that could be construed as a potential conflict of interest.
